# Direct Pars Defect Tubular Decompression and TLIF for the Treatment of Low-Grade Adult Isthmic Spondylolisthesis: Surgical Challenges and Nuances of a Muscle-Sparing Minimally Invasive Approach

**DOI:** 10.1155/2020/5346805

**Published:** 2020-10-31

**Authors:** Fabio Roberti, Katie Arsenault

**Affiliations:** ^1^Section of Neurosurgery, Cleveland Clinic Indian River Hospital, Vero Beach, FL, USA; ^2^Department of Neurosurgery, The George Washington University, Washington, DC, USA

## Abstract

We present an illustrative report on the use of a minimally invasive, muscle-sparing, direct pars defect decompression with transforaminal lumbar interbody fusion (TLIF) and instrumentation for the treatment of low-grade adult isthmic spondylolysis with spondylolisthesis and discuss the surgical challenges and nuances associated with the technique.

## 1. Introduction

Lumbar spondylolysis, an osseous congenital or acquired defect of the pars interarticularis, is a known cause of progressive low back pain in young patients. Repetitive stress and serial lumbar microtraumas involving the isthmus of the lumbar vertebrae explain the high prevalence of such condition among young athletes and patients with a documented history of competitive sport activities [1].

In adults or elderly patients with a history of chronic back pain, isthmic spondylolysis is often associated to symptomatic spondylolisthesis at the affected level. Mechanical spinal instability and debilitating radicular symptoms may also be present, and when conservative managements fails, surgical treatments may be highly effective in relieving severe symptoms and restoring a more physiological spinal alignment.

Although minimally invasive direct surgical treatments for lumbar spondylolysis have been well described [1–3] and minimally invasive instrumented fusion techniques are nowadays often selected to treat patients with lumbar spondylolisthesis [4–10], we believe that the addition in the literature of short illustrative surgical reports specifically addressing the surgical nuances and technical challenges associated with minimally invasive direct pars fracture decompression for isthmic spondylolysis with spondylolisthesis could be of benefit to surgeons in training and surgeons in the early stages of their minimally invasive surgery learning curve. The presence of fibrous pseudo-healing near the fracture line may in fact pose a surgical anatomy challenge that is not seen in degenerative spondylolisthesis, especially if combined to the relatively limited exposure allowed by a tubular approach.

We therefore present a report with intraoperative images of two cases (grade 1 and 2 isthmic spondylolisthesis) where such technique was adopted and discuss surgical challenges that may be encountered during this approach.

## 2. Operative Technique

Under general anesthesia, patients are positioned prone on a radiolucent Jackson table. In the presented cases, intraoperative lateral and anteroposterior (AP) fluoroscopy is utilized to guide the approach, but, if available, neuronavigation-assisted techniques could also be utilized to minimize X-rays' exposure [11].

Using intraoperative fluoroscopy, Jamshidi needles are carefully advanced bilaterally, avoiding any cortical breach, within the L5 and S1 pedicles. *K* wires are then passed to reach the posterior aspect of the vertebrae body and secured to the surgical sterile drape.

**Figure 1:**
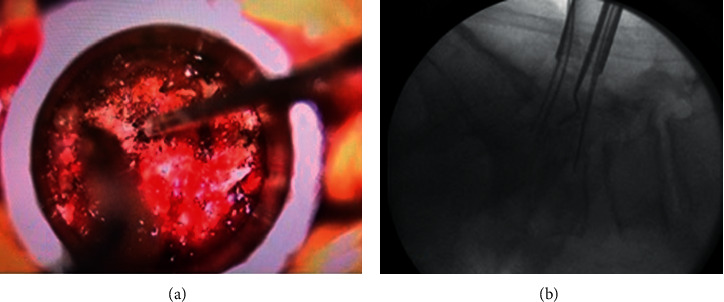
Grade 1 L5-S1 spondylolisthesis with the bilateral pars defect. (a) Intraoperative visualization of left L5 pars fracture (horizontally oriented). (b) X-ray confirmation of the pars fracture location (after placement of pedicles, *K* wires, and tubular exposure).

A tubular retractor (METRx, Medtronic) is then utilized to expose the posterior joint, as described in previous publications [12, 13]. The pars defect/fracture is then visualized just medial and superior to the facets, and radiological confirmation is obtained (Figure 1).

**Figure 2:**
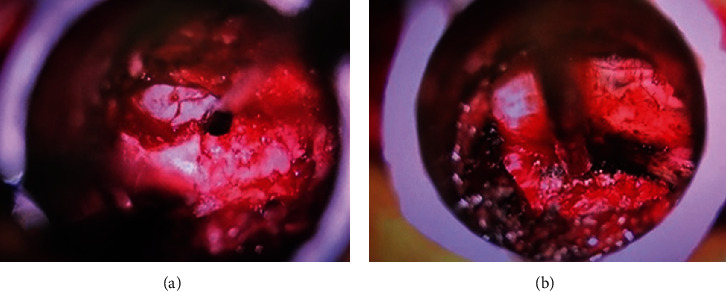
(a) The dura is localized at the beginning of a left L5 hemilaminectomy (the surgical instrument is placed over the medial aspect of the pars defect and fibrocartilaginous tissues). (b) Dura and decompressed left exiting (above the instrument) and left traversing (below the instrument) nerve roots are visualized.

**Figure 3:**
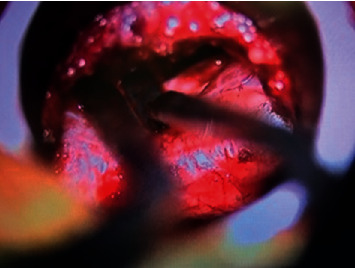
Contralateral nerve roots visible after bilateral decompression and central “over-the-top” laminectomy.

The fibrocartilaginous tissues surrounding the pars defect are removed, and with the use of a drill and Kerrison rongeurs, a L5 laminectomy is performed. Localization of the dura at this point of the procedure does facilitate surgical orientation in a field where common anatomical landmarks may be distorted and modified by the pathology at hand (Figure 2). Depending on the degree of central stenosis, an “over-the-top” tubular decompression may be carried out, so as to decompress the dura centrally and contralaterally [14–18]. If a central decompression is needed, we prefer to perform it near the end of the procedure so that, in case of CSF leak, the repair is easier, and no significant time is added to the duration of the surgery. Once the hemilaminectomy is completed, and the dura and traversing nerve roots localized, a foraminotomy over the traversing nerve root (S1, in a L5-S1 spondylolisthesis) is carried out. Next, the pars defect is removed, from medial to lateral, by the gentle use of a drill or using Kerrison rongeurs. Particular care should be taken during this step not to damage the exiting nerve root (L5 in a L5-S1 spondylolisthesis) while removing the cranial aspect of the pars fracture. The root is usually located just underneath the pars defect, with minimal or no surrounding epidural fat or ligament protecting it. X-rays' guidance and confirmation should be utilized during this step, if in doubt. Once the exiting nerve root is localized, the decompression can proceed under direct microscopic or loupe magnification until the dura, and exiting and traversing nerve roots are successfully decompressed (Figure 2). These steps are then repeated contralaterally, utilizing a second tubular retractor/arm, leaving in place the initial tubular retractor for convenience as well as to monitor hemostasis. Once the decompression has been completed bilaterally, the residual lamina underneath the spinous process (still attached to its ligaments) may be trimmed further thus completing “over-the-top” decompression. This maneuver will allow visualization of the contralateral neural elements (Figure 3).

**Figure 4:**
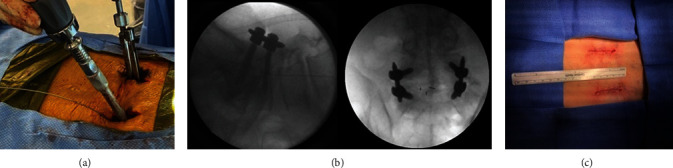
(a) Minimally invasive instrumented TLIF after bilateral pars decompression. (b) Final X-rays. (c) Wound closure.

**Figure 5:**
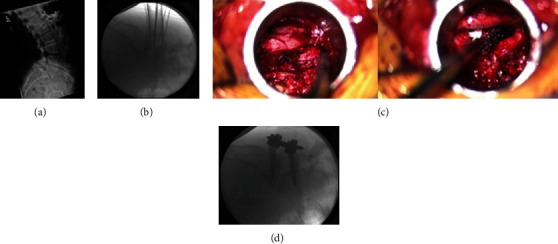
Grade 2 L5-S1 spondylolisthesis with the bilateral pars defect. (a) Preoperative X-ray. (b) Intraoperative localization during minimally invasive pars defect decompression. (c) Intraoperative visualization of decompressed exiting and traversing right nerve roots before and after TLIF. (d) Final lateral X-ray.

At this point, a minimally invasive TLIF with bilateral percutaneous instrumentation can be performed (usually from the most symptomatic side or, in case of bilateral symptoms, from the left side for right-handed surgeons). The use of an interbody graft will add indirect foraminal decompression, while posterior instrumentation may allow the possibility to reduce the listhesis in selected cases. Such TLIF is performed following the known technical steps previously described elsewhere [19, 20]. Once hemostasis is achieved, the fascia and wounds are closed bilaterally, and final X-rays are obtained (Figure 4). Intraoperative images of decompressed right neural elements (instead of left) of a grade 2 listhesis are also added to this report for completion of the presentation (Figure 5).

In the presented cases, no postoperative bracing was utilized, and patients were mobilized in the immediate postoperative period. Blood loss was <50 cc, and no complications were recorded. Radicular symptoms regressed quickly, and preoperative low back pain was significantly ameliorated at the 3- and 6-month follow-ups.

## 3. Discussion

Isthmic spondylolysis or pars interarticularis fracture, a bony defect most commonly located at L5, can be the source of chronic and debilitating back pain in young patients. The presence of such bilateral defect in an immature spine has been shown to carry a high risk of slow progression to spondylolisthesis as the patient ages, with the rate of progression slowing each decade. Unilateral defects, on the contrary, do not seem to be associated to a risk of slippage over the years [21]. The presence of a pars bony defect is usually diagnosed with conventional lateral and oblique-view lumbar X-rays, with multislice CT with multiplanar reconstruction and lumbar MRI being very useful in detecting the isthmic gap or bone marrow edema surrounding the fracture lines.

The presence of spondylolisthesis associated to a spondylolysis is a relatively common finding in adults or elderly patients that present with chronic low back pain and radicular signs/symptoms. When conservative treatments fail or in case of severe and progressive motor deficits, a surgical intervention is usually recommended. Direct posterior decompression, stand-alone indirect anterior decompression, and posterolateral arthrodesis with or without instrumentation, as well as 360° fusions, have all been utilized with success to treat these patients, and while current guidelines do not favor one approach over another, the addition of posterolateral fusion and 360° arthrodesis has shown to improve the clinical outcome in adult patients with low-grade (grade 1-2) isthmic spondylolisthesis. Circumferential fusions may also lead to higher radiographic arthrodesis rates, if compared to posterolateral fusion alone, in the same cohort of patients [22].

The use of minimally invasive techniques in spine surgery has shown to be effective in reducing length of stay, estimated blood loss, and use of postoperative narcotics, without compromising overall surgical outcome [23, 24]. Muscle injury can also be minimized by the use of minimally invasive muscle-sparing techniques [25], and selected minimally invasive procedures have also proven to be cost-effective in recently published cost-utility analysis [26, 27]. Although the use of minimally invasive techniques has grown over the past few years, now becoming part of the surgeons' armamentarium for the treatment of numerous spinal conditions [28–33], the number of technical reports and publications focusing on the surgical challenges associated with minimally invasive muscle-sparing treatment of isthmic spondylolysis with symptomatic spondylolisthesis in adults is still limited [34].

Like it is the case for many innovative surgical techniques, there is a learning curve associated with mastering and becoming proficient in the use of minimally invasive approaches to the spine [33]. Due to tailored but, sometimes, limited surgical exposure, need for dedicated instrumentation and distortion of the spinal anatomy by the pathology may all add levels of difficulty to the surgical procedure that can, at times, become a challenge even for experienced spine surgeons.

The presence of an isthmic fracture with chronic fibrous pseudo-healing and spondylolisthesis can make the recognition of surgical anatomy and orientation within the surgical field particularly difficult at times as the tubular approach only allows a relatively limited range of anatomical exposure and visualization. This may be especially the case during revision spine surgery or when a high degree of vertebral slippage is present. We did not utilize such technique to treat higher-grade spondylolisthesis (grade 3 or 4) as the minimally invasive exposure in such cases may be challenging, and we prefer, when possible, to obtain indirect foraminal decompression via an anterior interbody allograft fusion before performing a posterior decompression (with and without percutaneous instrumentation).

To facilitate the recognition of the surgical anatomy, the appropriate use of intraoperative fluoroscopy (or neuronavigation where available) is strongly encouraged.

Lateral X-rays should be utilized during the exposure to confirm localization and lumbar anatomy. In cases of revision surgery, where the spinous process and lamina had been previously removed, anteroposterior (AP) X-rays prove to be particularly useful to direct the tubular approach. In such redo surgeries in fact, it is not uncommon to err toward the contralateral side while exposing the superficial and scar tissues with the tubular retractor as the barrier provided by the spinous process may not be present anymore. Correct interpretation of an intraoperative AP X-ray can help correcting the angles of approach before the neural structures are encountered, thus facilitating the surgical orientation when needed. Radiation exposure for the patient, surgeon, and team can be minimized by stepping away from the surgical field, using protective gears, and applying the ALARA concept [35]. In the presented cases, only a combined average of 50 seconds of pulse radiation was sufficient to safely guide an approach that lasted approximately 180 minutes.

Once the lamina and pars fracture are localized, the line of fracture may be dissected from the surrounding fibrous tissues, and its orientation matched with preoperative images. Such line of fracture tends to be more horizontal than the lumbar facet joints (see Figure 1) and is always found superior to it (pars interarticularis). Once the fracture has been located and confirmed, surgical removal of the pseudo-joint and neural decompression can proceed, using the known microsurgical techniques, under direct loupe or microscope visualization. Particular care should be taken while decompressing the exiting nerve root at the level of the spondylolisthesis, which is usually located directly underneath the pars fracture. In light of the chronic nature of this condition and the usually severe degree of foraminal stenosis, the nerve root may be flattened against the bone with no significant surrounding epidural fat, and therefore, it may be prone to surgical injury during these steps. For this reason, we prefer to use a surgical drill over large Kerrisons while decompressing the foramen and performing the decompression. We recommend starting the dissection medially, performing a limited laminotomy, so that the lateral border of the dural sac can be visualized and used as the initial landmark (Figure 2).

Iatrogenic durotomy and subsequent CSF leak are not uncommon events during minimally invasive spine surgery [36].

Due to the tailored but limited exposure allowed by a tubular approach, the presence of a CSF leak during this procedure adds additional challenges and delays to the surgery. The deflation of the dural sac due to the loss of liquor may lead to venous oozing from the epidural plexus. The presence of blood in the operative field next to a durotomy may increase the risk of adhesive arachnoiditis, and, in some instances, the cauda equina rootlets may be protruding from the dural breach, exposing these neural elements to involuntary injury. Direct coagulation of the plexus may lead to further shrinkage of the dura resulting in worsening of the venous oozing, while the use of hemostatic agents directly over a durotomy is to be avoided. In such cases, we found it is useful to temporarily repair the leak (with some fat and fibrin glue) so that the dural sac can gradually re-expand thus facilitating a good hemostasis. The procedure is then completed working around the repair, which is then reinforced or redone before the final closure. Although using appropriate curved instruments and knot pushers, a direct dural suturing is possible, in our experience, we found this step is hardly needed. Due to the muscle-sparing nature of the tubular dissection, the risk of having a CSF leak above the fascia is very minimal as the paraspinal muscles tend to close the minimal surgical defect once the retractor is removed. If a direct dural suture is performed and more craniocaudal exposure is needed, the retractor system can be replaced with an expandable tubular retractor (X-tube, Medtronic) using the same skin and fascia incision by simply placing the larger dissection tube from the MetRx set inside the 22 mm tubular retractor and “redocking” the system before swapping the fixed retractor with the expandable device. Placing the interbody allograft before completing an “over-the-top” full laminectomy with contralateral decompression may also avoid the challenges associated with having to retract a deflated dura as it may be the case when a CSF leak occurs during such decompression.

## 4. Conclusion

We presented an illustrative report describing the use of minimally invasive tubular pars defect direct decompression with TLIF for the treatment of grade 1 and grade 2 symptomatic isthmic spondylolysis with spondylolisthesis in young adults. We discussed surgical challenges and nuances of the approach. In the presented cases, direct and indirect neural decompression, arthrodesis, and instrumentation were performed while maintaining the benefits associated to a muscle-sparing surgery. Although this well-described technique has gained a widespread use among spine surgeons, follow-up studies are needed to assess the long-term effectiveness of these less-invasive procedures in comparison to more traditional approaches. Surgeons interested in the minimally invasive treatment of such conditions should be aware of the particular nuances associated with the technique and allow themselves time to complete an appropriate and necessary learning curve. The correct use and interpretation of intraoperative X-rays (or neuronavigation) can be of great help in overcoming some of the challenges posed by the limited surgical exposure during minimally invasive spine surgery.
